# Single Crystals of Established Semiconducting Polymers

**DOI:** 10.3390/polym16060761

**Published:** 2024-03-10

**Authors:** Ioan Botiz

**Affiliations:** 1 Department of Physics of Condensed Matter and Advanced Technologies, Faculty of Physics, Babeș-Bolyai University, 400084 Cluj-Napoca, Romania; ioan.botiz@ubbcluj.ro; 2 Interdisciplinary Research Institute on Bio-Nano-Sciences, Babeș-Bolyai University, 400271 Cluj-Napoca, Romania

**Keywords:** semiconducting polymers, crystallization, single crystals, optoelectronic properties

## Abstract

In this work, we aim to deliver a comprehensive review of the past and current fabrication and subsequent structural characterization of single crystals of established semiconducting polymers and oligomers while maintaining extra emphasis on the crystals’ resulting optoelectronic properties, including charge carrier mobility, conductivity, photovoltaic capability, and the ability to absorb light.

## 1. Introduction

Semiconducting polymers owe their highly successful utilization in a plethora of state-of-the-art nanotechnological devices and interdisciplinary applications to their great optoelectronic attributes [1,2,3,4,5]. Nonetheless, these attributes heavily rely on not only their physical and chemical natures [6,7,8] but also on the microstructure–processing–property relationship [9,10,11] and can be tuned well by establishing precise control over the final polymer microstructure in solutions, thin films, or bulk [12]. Such control is therefore of paramount importance in the field of semiconducting polymers and can be achieved through the use of appropriate polymer-processing procedures [12].

There is a great multitude of processing methods used to control and tune the microstructures of semiconducting polymers as well as their corresponding chain conformations at nano- and microscales [12,13,14]. Most of these methods are commonly based on several fundamental physical ordering processes that include polymer phase separation [15,16,17,18], self-assembly [19,20,21,22], and crystallization [23,24,25,26]. While the process of self-assembly, for example, can deliver ordered semiconducting microstructures such as lamellae [22], cylinders [27], or other (spherical) assemblies [28], polymer crystallization (see discussions on the basic concepts of crystallization, including polymer diffusion, nucleation, and growth kinetics, elsewhere [29,30,31,32]) can generate high-quality single crystals of semiconducting polymers [25,33,34,35,36]. The latter represent highly ordered macromolecular assemblies made of semiconducting polymer chains that can be further regarded as model systems for a variety of (fundamental) studies [37,38,39]. This is because compared to bulk or any amorphous/semicrystalline microstructure, single crystals are long-range ordered macromolecular assemblies comprising polymer chains that all adopt a unique molecular conformation along three crystallographic axes (in the π-π stacking, side-chain lamellar packing, and backbone directions). Therefore, they contain no or few structural defects (such as weak lattice disorder caused by side-chain misalignments, paracrystallinity, or other defects [40]) and exhibit no grain boundaries. These properties favor fast/efficient charge transport through straightforward pathways within the crystals (for instance, one of the highest charge mobilities was demonstrated in high-quality single crystals [41]; this clearly justifies the need for single crystal fabrication when targeting improvements in the charge transport properties of semiconducting polymers) and are further responsible for other puzzling optoelectronic attributes such as increased exciton diffusion lengths, light absorption at longer wavelengths, and higher birefringence [25,30,37,39,42]. It is therefore not at all surprising to measure, for instance, exciton diffusion lengths as high as 200 nm in semiconducting polymer crystal-like nanofibers displaying well-ordered domains with increased molecular ordering and little energetic disorder [43].

Because semiconducting polymers have a stiffer backbone than most traditional flexible polymers, and because they can experience additional intermolecular interactions orthogonal to the backbone along the π-π stacking direction and along the side-chain direction, such polymers cannot fold as easily as their more flexible counterparts during the crystallization process and thus tend to adopt rather one-dimensional single-crystal structures [30]. Moreover, the crystallization kinetic process, which is based on nucleation and growth, is generally complex because it is highly dependent on various potentially limiting physical and chemical parameters like the chain length or polydispersity index that directly impact the entanglement of chains, chain diffusion, adsorption or desorption, and the exclusion of defects [44]. Furthermore, although many physical and chemical parameters that affect the crystallization of semiconducting polymers (i.e., dissolution and crystallization temperatures, the type of solvent, the polymer concentration, the type of polymer, nucleation and growth conditions, etc.) can be well-controlled [30], such polymer chains can become easily entangled; therefore, high free-energy barriers must be overcome in order to reorganize the chain conformations into ordered states when the polymer chains are processed from solution or melt phases [45]. Thus, fabricating single crystals of semiconducting polymers is not trivial but rather difficult [46], even though some single-crystal packing principles and preferences of semiconducting molecules are well known [47]. This is precisely why the literature on semiconductor polymer single crystals is rather scarce at the moment [48]. 

In this work, we aim to review both the internal structure and corresponding optoelectronic properties of single crystals of established semiconducting polymers mainly generated through processes such as crystallization (i.e., nucleation and growth) and to further emphasize the use of the resulting single crystals in practical applications. This means we will not discuss other methods of fabricating single crystals of semiconducting polymers that are based inclusively on polymerization reactions [49,50,51,52,53,54] (including topochemical polymerization in single-crystal-to-single-crystal transformations [55,56,57,58,59,60]) or on the use of two-dimensional (2D) and 3D covalent organic frameworks and the subsequent exploitation of various irreversible reactions [61] or dynamic covalent bonds [62,63,64,65,66] here. To find more about these polymerization-based single-crystal fabrication procedures, readers can consult other highly comprehensive reviews that were recently reported in the literature [30,44].

## 2. Fabrication and Structural Characterization of Single Crystals of Established Semiconducting Polymers 

### 2.1. Polythiophene-Based Single Crystals

Following Hermann’s 1930 “fringed micelle” model [67] and Storks’s 1938 idea of polymer chain folding within a lamellar crystal [68], polyethylene single crystals were already obtained and further studied in the middle of the 1950s by researchers like Jaccodine [69], Till Jr. [70], Fischer [71], and Keller [6]. Nonetheless, it was Keller who demonstrated that the molecular backbone was generally oriented along the thinnest dimension of the single polymer crystals; therefore, his “folded chain model” [6], which assumed the multiple folding of polymer chains within the crystal, was widely accepted by the scientific community. Moreover, in 1960, Hoffman and Lauritzen showed from a theoretical point of view how such polymer single crystals can form by developing the kinetics of the complex nucleation and growth processes [72].

**Figure 1:**
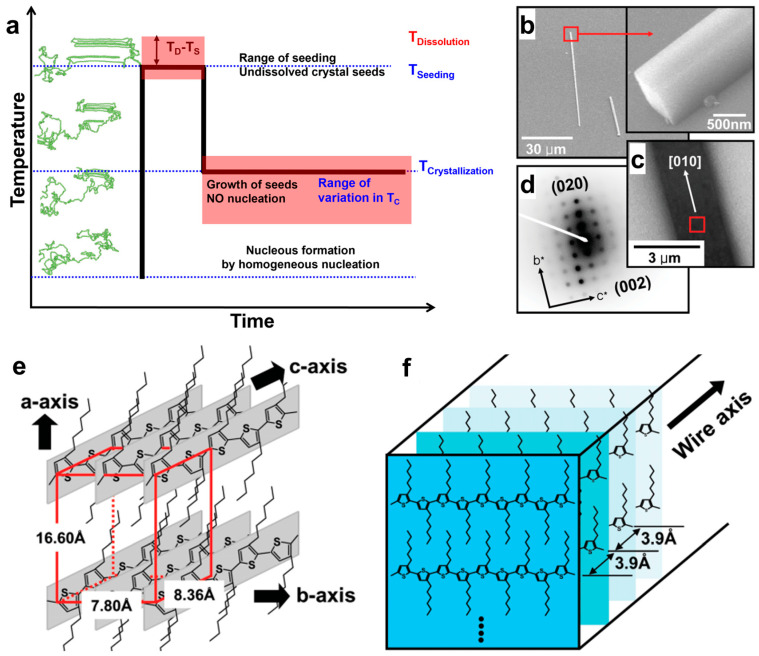
(**a**) Schematic depicting the self-seeding method, which generally comprises three successive main steps: (i) the dissolution of a specific polymer system in an appropriate solvent, followed by the subsequent formation of crystals/aggregates of different degrees of structural perfection, (ii) seed formation at a specific seeding temperature (*T_S_*), usually located (slightly) below the dissolution temperature (*T_D_*; at this temperature, all polymer molecules are considered to be homogeneously dissolved), and (iii) crystal growth at a lower crystallization temperature (*T_C_*) from the already existing seeds (no further nucleation takes place while the experiments are conducted in the range of *T_C_* variation). As a result, large single crystals can be obtained by precisely controlling *T_S_* and *T_C_*. (**b**) Field-emission scanning electron microscopy images of P3HT-single-crystal wires that were generated on an octadecyltrichlorosilane-covered silicon substrate. Here, the inset is displaying a side-view micrograph illustrating the rectangular cross section of the obtained P3HT microwires. (**c**) Transmission electron microscopy (TEM) image of P3HT single crystal wires on an octadecyltrichlorosilane-covered silicon nitride window, displaying the preferential growth of microwires along the [010] direction. (**d**) SAED pattern of P3HT single crystal microwires exhibiting diffraction equivalent to a repeating period of 0.78 nm along the π-π stacking direction (i.e., *b*-axis) and a periodicity of 0.836 nm along the chain (i.e., *c*-axis) direction. (**e**) A schematic representation of P3HT chains packed within the orthorhombic unit cell. (**f**) Schematics depicting the hierarchical self-assembly of P3HT chains into the reported single crystal microwires along the π-π stacking direction. Adapted from Ref. [34] (**b**–**f**) [copyright (2006) WILEY-VCH Verlag GmbH & Co. KGaA (Weinheim), with permission from John Wiley and Sons].

Currently, one of the most frequently studied classes of semiconducting polymers is the class of polythiophenes, with poly(3-hexylthiophene) (P3HT) being the most prominent member. Although the fabrication of crystals of oligothiophenes through a method based on physical vapor growth was reported in the late 1990s [73,74], the first single crystals obtained from P3HT systems were reported in 2006 by Kim et al., who employed the so-called self-seeding technique (see a description and schematic of this method depicted in Figure 1a) on silicon substrates covered with self-assembled octadecyltrichlorosilane monolayers to deliver one-dimensional (1D) microwire single crystals of a length of 30 to 500 µm. These crystals displayed an edge-on orientation, with the crystal length being oriented along the π-π stacking direction while the width direction was determined to be along the conjugated backbone (Figure 1b–f) [34]. Here, the authors employed a P3HT system with an average molecular weight of M_W_ = 54,000 g/mol dissolved in chloroform (the concentration was 0.1 mg/mL). This polymer solution was initially seeded at 40 °C (here, only crystalline/aggregated structures with a higher degree of order or a lower surface-to-volume ratio are expected to remain stable and thus to further act as seeds for immediate crystallization at a lower temperature) and then slowly cooled down to 10 °C, at which the crystallization process was conducted over three days. Selected area electron diffraction (SAED) patterns measured for the obtained single crystals showed Bragg diffraction spots (Figure 1d) and demonstrated the P3HT molecules packed in an orthorhombic crystal unit cell with the following lattice constants: *a =* 1.66 nm, *b* = 0.78 nm, and *c* = 0.836 nm (Figure 1e) [34].

**Figure 2:**
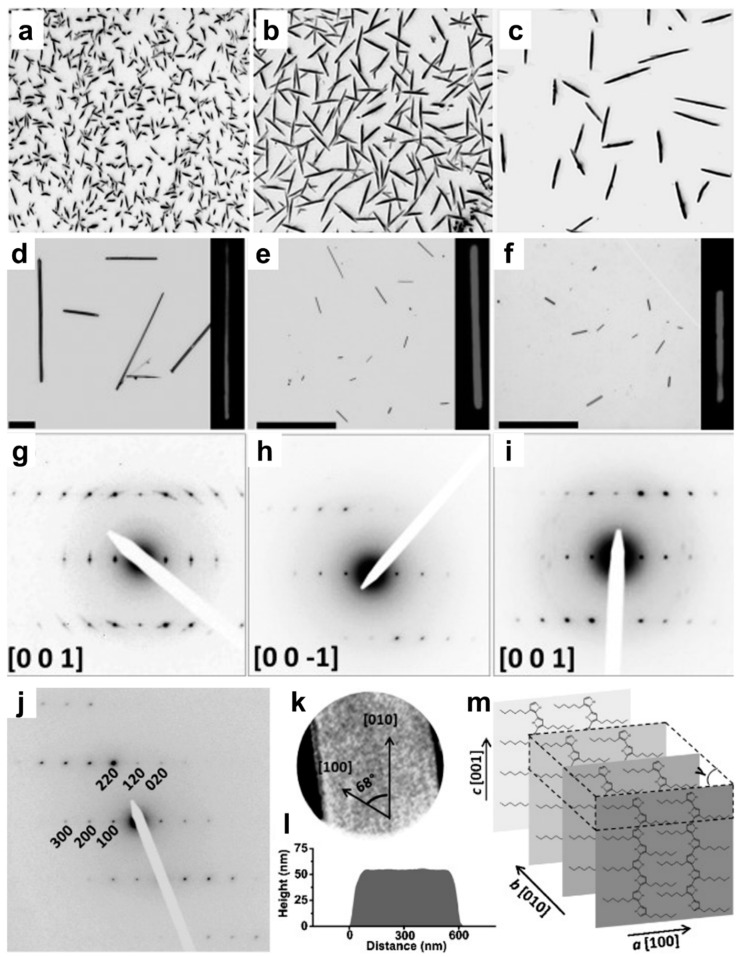
(**a**–**c**) Optical micrographs of single crystals of P3HT of M_n_ = 26,400 g/mol, obtained from 0.2 mg/mL primal solutions of 3-hexylthiophene that were seeded at 40 °C (**a**), 43 °C (**b**), and 47 °C (**c**) and then crystallized at 32 °C for 24 h before being spin-coated onto silicon substrates. All micrographs display an area of 100 × 100 μm^2^. (**d**–**f**) Optical micrographs depicting single crystals of (**d**) short P3HT (M_n_ = 1332 g/mol) grown in THF at 2 °C for 8 h, (**e**) P3HT of M_n_ = 3709 g/mol grown in 3-hexylthiophene at 10 °C for 12 h, (**f**) P3HT of M_n_ = 4700 g/mol grown in 3-hexylthiophene at 10 °C for 12 h. The scale bars shown in (**d**–**f**) represent 30 μm. The uniform brightness across the whole birefringent crystal shown in the right POM images of (**d**–**f**) further indicates the existence of highly ordered crystals comprising uniquely oriented P3HT chains. (**g**–**i**) SAED patterns recorded for P3HT single crystals depicted in (**d**–**f**), respectively. (**j**) SAED pattern measured for the single crystals of the longest P3HT macromolecule grown at 35 °C for 24 h after being previously seeded at 50 °C for 6 h. (**k**) A defocused diffraction pattern in the proper relative orientation to the electron diffraction pattern along with the [010] and [100] directions of the direct space unit cell axes. (**l**) The AFM height profile of a single crystal made of the longest P3HT macromolecule. (**m**) Schematics depicting the orientation of the longest P3HT macromolecules within the single crystal. Here, the size of the monoclinic unit cell is indicated by dashed lines. Adapted from Ref. [25] [copyright (2012) WILEY-VCH Verlag GmbH & Co. KGaA (Weinheim), with permission from John Wiley and Sons].

**Figure 3:**
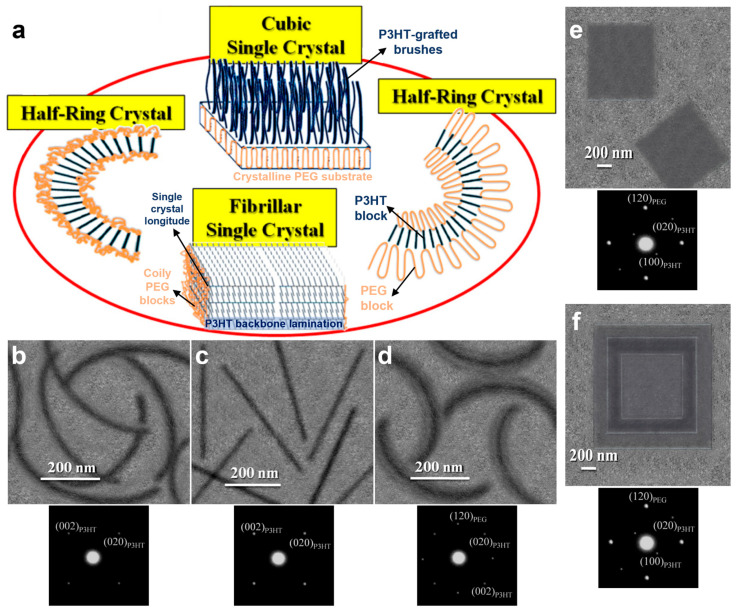
(**a**) Schematical representation of four different structures generated via the crystallization process. (**b**–**d**) TEM micrographs emphasizing the scrolled half-rings of a P3HT_7000_-*b*-PEG_5000_ block copolymer system that was isothermally crystallized at 30 °C (**b**), the nanofibers of a P3HT_7000_ homopolymer that were isothermally crystallized at 30 °C (**c**), and the scrolled half-rings of a P3HT_7000_-*b*-PEG_5000_ block copolymer system that crystallized under isothermal conditions at 10 °C (**d**). All three micrographs are accompanied by corresponding electron diffraction patterns placed as insets below the micrographs. (**e**,**f**) TEM micrographs displaying cubic single crystals that were seeded from a P3HT_7000_-*b*-PEG_5000_ block copolymer system and further grown at a crystallization temperature of 30 °C for 24 h in amyl acetate (**e**) and single crystals of PEG_5000_/P3HT_7000_-*b*-PEG_5000_/PEG_5000_ that were obtained by employing epitaxial growth (**f**). Again, these micrographs are accompanied by corresponding electron diffraction patterns placed as insets below the micrographs. Adapted from Ref. [75] [copyright (2016), with permission from the American Chemical Society].

Only few years, later two other research groups reported the preparation of single crystals of P3HT, this time displaying needle-like shapes [25,35]. In the first case, Xiao et al. fabricated P3HT single crystals from thin films by both employing tetrahydrofuran vapor annealing and establishing control over the evaporation of the solvent [35]. More precisely, a droplet of solution containing P3HT molecules dissolved in chloroform was drop-casted onto the substrate and generated a polycrystalline film. The latter was further placed and kept for different periods of time on top of an open container full of tetrahydrofuran (THF) generating THF vapors at 35 °C under ambient pressure. This ensemble was further sealed in an airtight container to optimize the process of solvent annealing. Once the residual THF solvent vapors were removed in a controlled fashion, single P3HT crystals exhibiting a length of 20 to 60 μm and a width of 1 to 2.2 μm were generated [35]. The resulting needle-like single crystals had the π-π stacking direction oriented perpendicularly to the length axis of the crystals, while the alkyl side chains were shown to orient parallel to the substrate [35]. Unfortunately, with this method, it was difficult to establish full control over the number of single crystals that were generated. This was not an issue anymore in the second case, when Rahimi et al. utilized the self-seeding technique to control the number density and size of needle-like P3HT single crystals produced from dilute solutions (Figure 2) [25]. The solutions, which were prepared by dissolving regioregular P3HTs of different molecular weights (M_n_ = 26,400 g/mol, M_n_ = 4700 g/mol, M_n_ = 3709 g/mol and M_n_ = 1332 g/mol) in a 3-hexylthiophene (or THF) solvent, were initially heated to specific seeding temperatures, *T_S_*, for 6 h and then kept at lower crystallization temperatures, *T_C_*, for various periods of time. Unsurprisingly, it was found that the size of the single P3HT crystals strongly depended on the *T_S_* (Figure 2a–c). For instance, processing primal P3HT solutions at a higher *T_S_* led to less crystalline/aggregated structures surviving from such solutions and thus generated fewer nuclei (seeds) that could further develop at a lower *T_C_* into larger and fewer P3HT single crystals (nonetheless, it was observed that all single crystals seeded at a specific *T_S_* displayed approximately the same size) [25]. Large P3HT single crystals with a length of more than 80 μm were obtained by varying both the *T_S_* and *T_C_*. Note here that while homogeneous nucleation was not observed for the high-molecular-weight system, it was used to grow P3HT single crystals of lower molecular weights (Figure 2d–f) as the primal crystalline/aggregated structures were rapidly dissolved and left behind no visible seeds when approaching the *T_D_*. SAED experiments (Figure 2g–j) demonstrated that the obtained single crystals had a monoclinic crystal structure of form II (the lattice parameters can be found in Table 1). Moreover, while the P3HT chains adopted a fully extended conformation and were orthogonally oriented with respect to the substrate, the side chains were fully interdigitated (Figure 2m). Furthermore, atomic force microscopy (AFM) measurements revealed that the obtained single crystals displayed heights ranging from ~59 nm (corresponding to the longest P3HT chains; Figure 2l) to 3 nm (corresponding to the shortest P3HT chains). Finally, all single crystals had their length direction oriented along the π-π stacking direction, indicating that the direction of faster growth was dictated by the π-π stacking process [25]. This highly ordered internal structure of the P3HT single crystals was further confirmed by their high birefringence, measured using optical microscopy with crossed polarizers (POM; right images in Figure 2d–f) [25] as well as by considering additional precise structural markers inferred from Raman vibrational spectra [39].

At the end of 2016, Agbolaghi et al. employed non-seeding and seeding protocols to fabricate several distinct types of P3HT-based single crystals (Figure 3a) [75]. On one hand, by taking advantage of the isothermal crystallization of a P3HT homopolymer and poly(3-hexylthiophene)-*b*-poly(ethylene glycol) (P3HT-*b*-PEG) block copolymers, they fabricated non-seeded edge-on-oriented fibrillar and scrolled half-ring-like crystals (Figure 3b–d). In these structures, while the P3HT chains adopted an extended chain conformation of about 17 nm, the PEG chains assembled into lamellae located on both sides of the P3HT half-rings, with the outer PEG lamella being thicker and wider than the inner one [75]. On the other hand, the authors used homogeneous seeding in polymer solutions based on tiny crystals made of either PEG_5000_ or P3HT_7000_ homopolymers [75]. In the former case, the developed cubic PEG_5000_ single crystals were further sandwiched between two layers of grafted P3HT_7000_ chains, leading to the growth of P3HT_7000_-*b*-PEG_5000_ cubic single crystals that exhibited a flat-on orientation of P3HT_7000_ backbones on the PEG_5000_ single crystalline substrate (Figure 3e). Moreover, more complex structures such as epitaxially grown PEG_5000_/P3HT_7000_-*b*-PEG_5000_)/PEG_5000_ single crystals could be grown from amyl acetate at 30 °C (Figure 3f). Here, the inner core of the epitaxial structure was a single crystal of PEG_5000_, previously seeded at 40 °C and then crystallized at 30 °C for 8 h, that was injected into a P3HT_7000_-*b*-PEG_5000_ solution and homogenized at 30 °C for 3 min. The crystallization of the whole PEG_5000_-crystal/P3HT_7000_-*b*-PEG_5000_ ensemble proceeded for another 8 h and finally led to channel wire structures composed of PEG_5000_ blocks and P3HT_7000_-grafted brushes (Figure 3f) [75]. Instead, in the latter case, the authors seeded the homogenized P3HT_7000_-*b*-PEG_5000_ solution using tiny P3HT_7000_ single crystals. This procedure led to the formation of edge-on-orientated fibrillar P3HT_7000_-*b*-PEG_5000_ and P3HT_7000_ single crystals of similar thicknesses and lateral dimensions but with very different backbone lamination along the *c*-axis caused by the fact that in the P3HT_7000_-*b*-PEG_5000_ single crystals, the P3HT_7000_ backbones of were not capable of laminating from the coily block sides [75].

Other examples of single crystals grown from P3HT-based block copolymers were reported more recently by Zenoozi et al. [76,77]. They employed isothermal crystallization for over 3 days to grow 57–139 nm thick single crystals and 2–8 nm thick nanofibers from a P3HT homopolymer as well as from poly(3-hexylthiophene)-*b*-polystyrene (P3HT-*b*-PS), P3HT-*b*-PEG and poly(3-hexylthiophene)-b-poly(methyl methacrylate) (P3HT-*b*-PMMA) dissolved in a variety of solvents, including toluene, xylene, and anisole [77]. Here, all single crystals, along with their building crystallites, were longer in the π-π stacking direction. Moreover, seeding at higher temperatures caused the single crystals and nanofibers and their corresponding building block crystallites to be thicker and longer, in line with findings reported elsewhere [25]. Furthermore, in these types of single crystals made of P3HT-based block copolymers, coily blocks such as PS, PEG, and PMMA were not part of the P3HT crystalline structures and thus generated some brush-like or hairy areas on the surface of the latter, leading to hairy single crystals [76]. Finally, one could also observe that larger single crystals generated from less good solvents at higher seeding temperatures displayed a flat-on orientation, with longer P3HT chains experiencing folding. More details on the internal structure characteristics of these single crystals and nanofibers can be found in Table 1.

**Table 1:** Summary of various polymer single crystals that were generated from established semiconducting polymers.

Polymer	Molecular Weight (g/mol)	Crystal Type	Crystal Length (µm)	*a*-Axis (nm)	*b*-Axis (nm)	*c*-Axis (nm)	Unit Cell	Ref.
P3HT	M_w_ = 54,000	microwire	500	1.66	0.78	0.836	orthorhombic	[34]
P3HT	M_w_ = 39,600	needle-like	60	1.636	0.838	0.742	-	[35]
P3HT	M_n_ = 26,400	needle-like	>80	1.31	0.93	-	monoclinic	[25]
P3HT	M_n_ = 4700	needle-like	~10	1.23	0.91	-	monoclinic	[25]
P3HT	M_n_ = 3709	needle-like	>15	1.26	0.92	-	monoclinic	[25]
P3HT	M_n_ = 1332	needle-like	>130	1.32	0.95	-	monoclinic	[25]
P3HT	M_n_ = 7000	nanofiber	~0.4	1.84	0.399	-	-	[75]
P3HT	M_n_ = 7000	fibrillar	60	1.681	0.364	-	-	[75]
P3HT	M_n_ = 7150	nanofiber	0.65 ^a^ 15.5 ^c^	1.95 ^c^	0.351 ÷ 0.367 ^a^ 0.413 ÷ 0.429 ^c^	-	-	[77]
P3HT	M_n_ = 7150	needle-like	>40 ^a^ >70 ^b^	1.343 ^a^ 1.603 ÷ 1.713 ^b^	0.33 ^a^ 0.374 ÷ 0.389 ^b^	-	-	[76,77]
P3HT	M_n_ = 21,000	nanofiber	0.55 ^a^	1.949 ^c^	0.351 ÷ 0.367 ^a^ 0.394 ÷ 0.408 ^b^ 0.413 ÷ 0.429 ^c^	-	-	[77]
P3HT	M_n_ = 21,000	needle-like	~68 ^b^ >128 ^c^	1.342 ^a^ 1.605 ^b^ 1.850 ÷ 1.958 ^c^	0.330 ^a^ 0.374 ÷ 0.389 ^b^ 0.4 ÷ 0.413 ^c^	-	-	[76,77]
P3HT	M_n_ = 48,800	nanofiber	0.45 ^a^	1.95 ^c^	0.351 ÷ 0.367 ^a^ 0.394 ÷ 0.408 ^b^ 0.413 ÷ 0.429 ^c^	-	-	[77]
P3HT-*b*-PEG	M_n_ = 12,000	scrolled half-rings	~0.5	1.845	0.408	-	-	[75]
P3HT-*b*-PEG	M_n_ = 12,000	cubic	0.91	1.991	0.424	-	-	[75]
P3HT-*b*-PEG	M_n_ = 12,000	fibrillar	63	1.68	0.364	-	-	[75]
PEG/P3HT-*b*-PEG/PEG	M_n_ = 5000 M_n_ = 12,000	epitaxial channel wire/cubic	~1.5	1.99	0.425	-	-	[75]
P3HT-*b*-PEG	M_n_ = 7900	hairy nanofiber	>9 ^b^	1.502 ÷ 1.617 ^a^ 1.75 ÷ 1.869 ^b^ 1.948 ÷ 2.044 ^c^	0.351 ÷ 0.367 ^a^ 0.394 ÷ 0.408 ^b^ 0.413 ÷ 0.429 ^c^	-	-	[77]
P3HT-*b*-PEG	M_n_ = 7900	hairy needle-like	~40	1.464 ^a^ 1.713 ^b^ 1.958 ^c^	0.347 ^a^ 0.374 ÷ 0.389 ^b^ 0.4 ÷ 0.413 ^c^	-	-	[76,77]
P3HT-*b*-PEG	M_n_ = 21,750	hairy needle-like	32	1.397 ^a^ 1.667 ^b^	0.341 ^a^	-	-	[76,77]
P3HT-*b*-PEG	M_n_ = 49,550	nanofiber	0.98 ^a^	1.512 ^a^	0.356 ^a^ 0.394 ÷ 0.408 ^b^ 0.413 ÷ 0.429 ^c^	-	-	[77]
P3HT-*b*-PEG	M_n_ = 49,550	hairy needle-like	~148 ^c^	1.351 ^a^	0.33 ^a^	-	-	[76,77]
P3HT-*b*-PS	M_n_ = 7669	hairy nanofiber	1.79 ^a^ >9 ^b^	1.862 ^b^ 2.036 ^c^	0.351 ÷ 0.367 ^a^ 0.405 ^b^ 0.413 ÷ 0.429 ^c^	-	-	[77]
P3HT-*b*-PS	M_n_ = 7669	hairy needle-like	~162 ^c^	1.456 ^a^ 1.705 ^b^	0.343 ^a^ 0.385 ^b^ 0.4 ÷ 0.413 ^c^	-	-	[76,77]
P3HT-*b*-PS	M_n_ = 21,519	hairy needle-like	82 ^b^	1.391 ^a^ 1.661 ^b^ 1.850 ÷ 1.958 ^c^	0.33 ÷ 0.347 ^a^ 0.374 ÷ 0.389 ^b^ 0.4 ÷ 0.413 ^c^	-	-	[76,77]
P3HT-*b*-PS	M_n_ = 49,319	hairy nanofiber	13.5 ^c^	1.95 ^c^	0.351 ÷ 0.367 ^a^ 0.394 ÷ 0.408 ^b^ 0.413 ÷ 0.429 ^c^	-	-	[77]
P3HT-*b*-PMMA	M_n_ = 7647	hairy nanofiber	9.38 ^b^ 21.38 ^c^	1.502 ÷ 1.617 ^a^ 1.75 ÷ 1.869 ^b^ 1.948 ÷ 2.044 ^c^	0.351 ÷ 0.367 ^a^ 0.394 ÷ 0.408 ^b^ 0.413 ÷ 0.429 ^c^	-	-	[77]
P3HT-*b*-PMMA	M_n_ = 7647	hairy needle-like	~49 ^a^ ~95 ^b^ >21 ^c^	1.342 ÷ 1.464 ^a^ 1.603 ÷ 1.713 ^b^ 1.850 ÷ 1.958 ^c^	0.33 ÷ 0.347 ^a^ 0.374 ÷ 0.389 ^b^ 0.4 ÷ 0.413 ^c^	-	-	[77]
P3HT-*b*-PMMA	M_n_ = 21,497	hairy nanofiber	1.15 ^a^ >9 ^b^	1.502 ÷ 1.617 ^a^ 1.75 ÷ 1.869 ^b^ 1.948 ÷ 2.044 ^c^	0.351 ÷ 0.367 ^a^ 0.394 ÷ 0.408 ^b^ 0.413 ÷ 0.429 ^c^	-	-	[77]
P3HT-*b*-PMMA	M_n_ = 49,297	hairy needle-like	~17.5 ^c^	1.342 ÷ 1.464 ^a^ 1.603 ÷ 1.713 ^b^ 1.850 ÷ 1.958 ^c^	0.33 ÷ 0.347 ^a^ 0.374 ÷ 0.389 ^b^ 0.4 ÷ 0.413 ^c^	-	-	[77]
P3BT	M_w_ = 16,000	needle-like	>1000	1.42	2.35	1.56	-	[78]
P3OT	M_w_ = 120,000	needle-like	~50	-	0.838	0.742	-	[36]
P3OT	M_w_ = 51,200	rod-like	~50	-	0.838	0.742	-	[35]
PDTTDPP	-	nanowire	<40	1.92	0.37	2.12	orthorhombic	[79]
DPPBTSPE	M_n_ = 8000	nanowire	100	1.898	0.346	2.034	orthorhombic	[80]
DPPBTSPE	M_n_ = 68,000	nanowire	>50	1.898	0.346	2.034	orthorhombic	[80]
TA-PPE	M_w_ = 51,328	nanowire	tens	1.363	0.762	0.512	orthorhombic	[81]
CDT-BTZ	M_n_ = 50,000	fiber	20	-	0.37	-	-	[82]
F16	M_MS_ = 6220	rod-like ^d^ lenticular ^e^ fibrous ^f^	200 ~2 several	2.16 ^d^	1.28 ^d^	3.36 ^d^	orthorhombic ^d^	[33,83]
F32	M_MS_ = 12,437	rod-like ^d^ lenticular ^e^ fibrous ^f^	>10 ~2 several	2.16 ^d^	1.28 ^d^	3.36 ^d^	orthorhombic ^d^	[33,84]
F64	M_MS_ = 24,874	rod-like ^d^ lenticular ^e^ fibrous ^f^	>10 ~2 several	2.16 ^d^	1.28 ^d^	3.36 ^d^	orthorhombic ^d^	[33,83,84]
Fn	M_n_ = 100,957	rod-like ^d^	~24	-	-	-	-	[85]
TANI	-	plate-like	several	0.68	0.78	2.4	-	[86]
F4BDOPV-2T	M_n_ = 60,400	microwire	~100	3.127	0.412	2.485	triclinic	[87]
P(NDI2OD-T2)	M_n_ = 76,600	microwire	~50	2.723	0.451	1.418	triclinic	[87]

M_n_ = number average molecular weight; M_w_ = weight average molecular weight; M_MS_ = molecular weight; ^a^ = in toluene; ^b^ = in xylene; ^c^ = in anisole; ^d^ = in chloroform/ethanol (or toluene/ethanol, ratio 1:1); ^e^ = in toluene; ^f^ = in toluene/ethanol (ratio 3:1).

Other important members of the polythiophenes class that were employed to fabricate single crystals are poly(3-butylthiophene) (P3BT) and poly(3-octylthiophene) (P3OT) systems [35,36,78]. In the first case, Ma et al. used a head-to-tail regioregular P3BT (M_w_ = 16,000 g/mol) to develop single crystals with a flat-on lamellar orientation and extended chain conformations by employing the solvent-assisted crystallization method [78]. More precisely, the initial P3BT films, prepared by drop-casting from a THF solution onto a solid substrate and followed by slow solvent evaporation, were further introduced into a mixture of nitrobenzene and THF for a couple of days until the solvents evaporated completely. This process led to P3BT single crystals exhibiting a lamellar structure (here, the main polymer chains were packed normal to the substrate, while the alkyl side chains were displayed along the direction of crystal growth), with a lamellar thickness ranging between about 15 nm to over 100 nm [78]. In the second case, Xiao et al. took advantage of the Ostwald ripening process induced by solvent vapor annealing in THF and generated from initial P3OT polycrystalline films needle-like single crystals with a conjugated backbone of P3OT chains (M_w_ = 120,000 g/mol) displayed along the long axis of the crystal (while the alkyl side chains were orthogonally oriented with respect to the substrate) [36]. These results, along with data presented in Table 1, were inferred from optical (OM), transmission (TEM), and scanning (SEM) electron microscopy measurements, as well as SAED and X-ray diffraction (XRD) patterns. Similar single crystals were also obtained when exposing shorter P3OT chains (M_w_ = 51,200 g/mol) to solvent vapor annealing in THF under controlled solvent evaporation conditions [35]. In this case, while the main P3OT chains adopted a parallel arrangement with respect to the substrate, these macromolecules were arranged with the π-π stacking direction parallel to the length axis of the crystal [35].

Recently, single crystal nanowires of a thiophene-based copolymer, made of diketopyrrolopyrrole (DPP) and dithieno [3,2-b:20,30-d]thiophene (DTT) and abbreviated PDTTDPP, were self-assembled by Kim et al. by simply storing the dissolved and previously filtered PDTTDPP solution at room temperature for a period of several days [79]. The formation of nanowires was favored by the rigid and planar DTT, which improved the interchain interactions and further stimulated the assembly process of the PDTTDPP backbones. SAED measurements showed that the PDTTDPP single crystal nanowires were highly crystalline and displayed an orthorhombic crystal structure, with the polymer chain axis being parallel to the longitudinal axis of the wire, while the π−π stacking direction was orthogonal with respect to the wire growth direction [79]. Even more recently, Um et al. first synthetized high- and low-number-average-molecular-weight thiophene samples containing polymers (DPPBTSPE) from 1,2-bis(5-(thiophen-2-yl)selenophen-2-yl)ethene) and then fabricated single-crystal nanowires via a method based on self-assembly [80]. The nanowires obtained from longer polymer chains exhibited a higher aspect ratio. From SAED and 2D grazing incidence X-ray diffraction (2D GI-XRD) measurements, it was inferred that the obtained single crystal nanowires had an orthorhombic crystal structure, with the polymer chains not only aligned parallel to the long axis of the nanowires but also packed densely and occupying the smallest volume in the unit cell [80]. Highly crystalline nanowires and fibers were also reported earlier for other types of semiconducting polymers such as rigid, rod-like poly(*para*-phenylene ethynylene)s based on thioacetate end groups (TA-PPE) [81] or cyclopentadithiophene–benzothiadiazole (CDT-BTZ) donor–acceptor copolymers [82]. In the former case, Wang et al. generated, by employing a method based on slow self-assembly from dilute solutions, high-quality crystalline nanowires displaying single-crystal orthorhombic diffraction patterns (with the polymer backbone arranged parallel to the nanowire long axis and with their side chains packed orthogonal with respect to the substrate) [82]; in the latter example by using solvent-vapor-enhanced drop-casting, high-molecular-order quasi-crystal-like single fibers exhibiting a strong alignment of copolymer backbones along the fiber axis were produced [82].

### 2.2. Polyfluorene-Based Single Crystals

**Figure 4:**
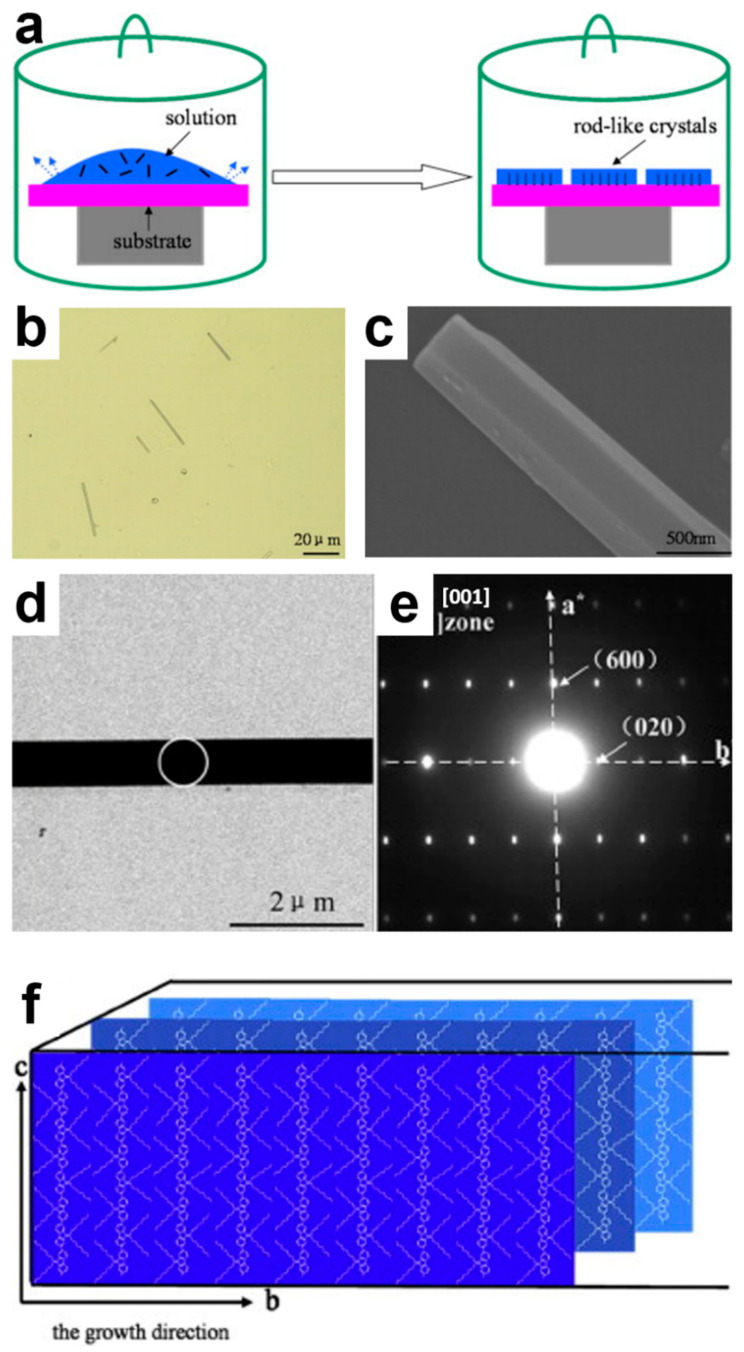
(**a**) Schematical representation of the method used to slowly grow single crystals of F16, F32, and F64. (**b**) Optical micrograph depicting F16 single crystals. (**c**) SEM image zooming in on an F16 single crystal. (**d**) Bright-field electron microscopy image of an F16 single crystal displaying preferential growth along the [010] direction. (**e**) SAED pattern recorded for the area emphasized in (**d**) by the white circular shape. (**f**) Simplified schematics showing the lamellar packing of PFO chains in the obtained single crystals. Adapted from Ref. [33] [copyright (2013), with permission from Elsevier Ltd.].

Another interesting class of semiconducting polymers used to fabricate single crystals is the class of polyfluorenes (PFOs) [33,83,84,85]. Liu et al. have utilized monodisperse poly(9,9-dioctylfluorene)s (PFOs) such as F16 (with a molecular weight of M_MS_ = 6220 g/mol), F32 (M_MS_ = 12,437 g/mol), F64 (M_MS_ = 24,874 g/mol), and Fn (M_n_ = 100,957 g/mol), either neat or in mixed components, to grow rod-like lamellar single crystals comprising extended chains packed into orthorhombic unit cells [33] (Figure 4). Interestingly, while the PFO backbones were orthogonally oriented with respect to the lamellar surface, the alkyl side chains were oriented along the direction of crystal growth (Figure 4f) [33]. The demonstrated possibility of controlling the morphology of PFO crystals and obtaining various crystalline PFO forms by tuning the good/poor solvent ratio in the solvent mixture of toluene and ethanol was also puzzling [84]. With this methodology, additional lenticular and fibrous crystals (see their properties in Table 1) could have been produced via slow crystallization that forced the PFO chains to undergo different kinetic pathways [84]. Crystallization was conducted using diluted chloroform/ethanol solutions that were deposited onto solid substrates and further exposed to thermal annealing under a sealed nitrogen atmosphere (Figure 4a) [83]. Moreover, the process of crystallization involving these PFO systems was accompanied by fractionated crystallization. Here, the saturation solubility of various components greatly affected the outcome of the crystallization process [83,85]. Furthermore, it was demonstrated that the edge-on orientation of the rod-like PFO single crystals could be adjusted to a flat-on orientation by tuning the type of solvent [84].

### 2.3. Single Crystals of Other Semiconducting Oligomers/Polymers

**Figure 5:**
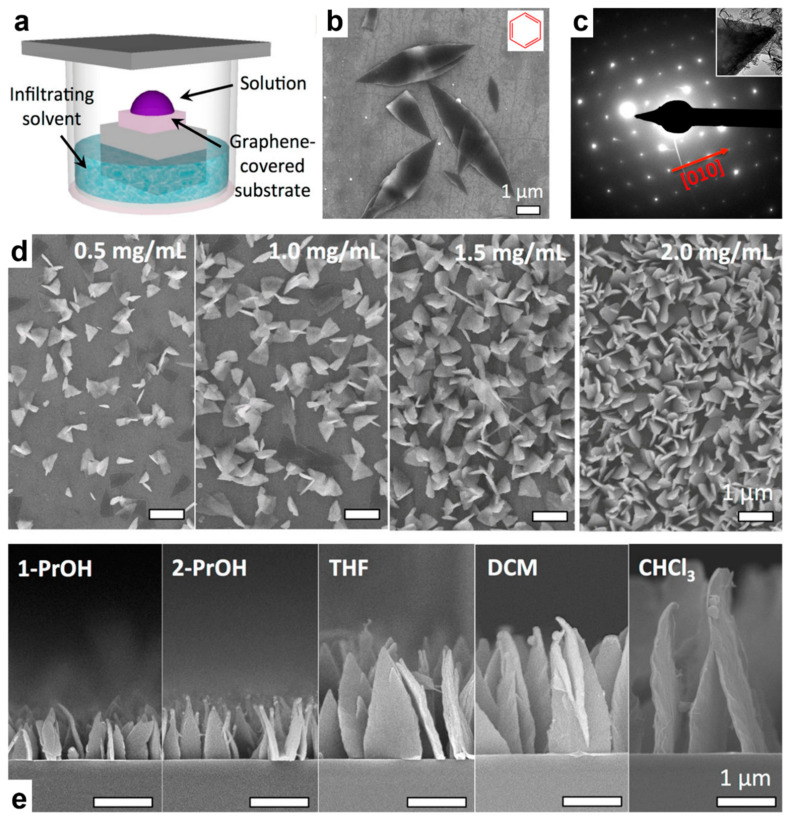
(**a**) Schematics depicting the vapor infiltration method employed to grow TANI single crystals. (**b**) Horizontal TANI single crystals produced by utilizing benzene as the infiltrating solvent. (**c**) The SAED pattern for the TANI single crystal shown in (**b**). (**d**) Top-view SEM images depicting the correlation between the nucleation density of vertical single crystals and the TANI concentration used. (**e**) Side-view SEM images emphasizing the correlation between the size of the TANI single crystals and the quality of the utilized solvents at a constant TANI concentration of 2 mg/mL. Adapted from Ref. [86] [copyright (2015), with permission from the American Chemical Society].

This section describing the production of all types of single crystals based on semiconducting polymers will end with two final examples. In the first example, a short oligoaniline (TANI) molecule was employed by Wang et al., who grew random and vertically oriented semiconducting single crystals by designing a controllable “bottom-up” method based on the utilization of graphene as a guiding substrate and a vapor-infiltration setup (Figure 5a) [86]. More precisely, they conducted all crystallization experiments in a covered Petri dish that was partly filled with a (non)solvent for TANI and contained an ensemble comprising a piece of a graphene-coated silicon wafer on top of a thick glass stage. TANI solutions from a variety of solvents were then drop-casted one at a time on the substrate while the vapors of the (non)solvent were saturating the Petri dish and then infiltrating the TANI solution, finally inducing supersaturation, nucleation, and single crystal growth via crystallization. All the single crystals produced displayed impressive morphological (Figure 5b) and crystallographic (Figure 5c) orientations which indicated that the TANI molecules were π-stacked orthogonally with respect to the graphene substrate (note that the TANI molecules were also π-stacked parallel to the graphene substrate and along the long axis of the single crystals when the single crystals were grown horizontally by employing an aromatic infiltrating solvent; Figure 5b) [86]. Moreover, by varying the TANI concentration in the solutions and tuning the quality of the utilized solvents, both the nucleation density and size of the single crystals could be precisely controlled (Figure 5d–e) [86].

The last example discusses the most recent production of crystals of relatively high electron mobility based on a semiconducting polymer, which was reported in 2021 by Yao et al. This research team grew crystal microwires of a four-fluorinated benzodifurandione-based oligo(p-phenylene vinylene) (F4BDOPV-2T) and naphthalenediimide containing P(NDI2OD-T2) polymers by employing the already well-known self-seeding growth procedure that consisted of polymer dissolution at 140 °C, followed by self-seeding and growth via slow cooling and ending with crystal growth at 25 °C [87]. Moreover, it was shown that the obtained F4BDOPV-2T microwire crystals had a length of ~100 μm and displayed strong diffractivity due to the highly ordered packing of polymer chains (powder X-ray diffraction experiments and additional molecular modeling based on density functional theory suggest that the F4BDOPV-2T chains were densely π-π stacked and had interdigitated alkyl chains; the simulated parameters of the triclinic unit cell were *a* = 3.127 nm, *b* = 0.412 nm, and *c* = 2.485 nm; see Table 1) [87]. Instead, the P(NDI2OD-T2) microwire crystals exhibited a shorter length of ~50 µm, with it suggested that their chains pack into a triclinic unit cell as well.

## 3. Optoelectronic Properties and Applications of Single Crystals of Established Semiconducting Polymers and Oligomers 

The optoelectronic properties of single crystals of established semiconducting polymers mainly dictate the suitability of certain material/crystals for specific targeted nanotechnological applications and include charge carrier mobility and conductivity, photovoltaic capability, and the ability to absorb light, to name the most important properties (a summary of such characteristics is listed in Table 2 and Table 3). In the next subsections, we will review the most significant examples that exist in the literature and report the optoelectronic characteristics of single crystals of semiconducting polymers/oligomers, with an extra emphasis on developed applications.

### 3.1. Charge Carrier Mobility in Single Crystals of Semiconducting Polymers

One of the most interesting properties of semiconducting polymer chains is their ability to transport charges along and in between them. Charge carrier mobility thus refers to the speed at which charge carriers such as electrons or holes can move in a (polymeric) material along a specific/given direction (most commonly along the π-π stacking direction or along the backbone direction) in the presence of an electric field [88]. 

**Figure 6:**
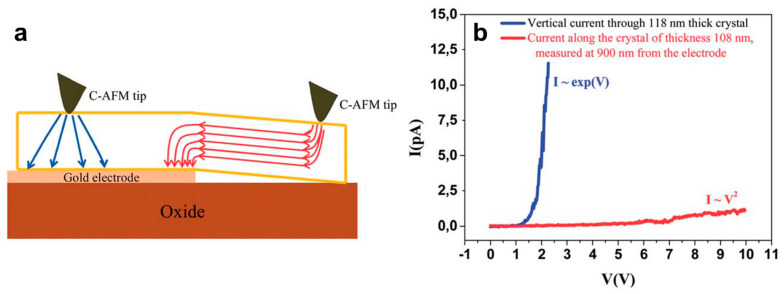
(**a**) Illustration of a single oligothiophene crystal placed on a gold electrode and further extending on an oxide substrate experiencing a vertical current in the direction of the molecular axis and a current along the π-π stacking direction (i.e., along the long axis of the crystal). (**b**) I–V curves measured both in the vertical direction of the single crystal (blue) and along the crystal at a distance of 900 nm from the gold electrode (red). Reproduced from Ref. [37]—published by The Royal Society of Chemistry.

Among all single crystals that were developed from P3HT-established polymers, we found two scientific reports in the literature in which needle-like crystals were evaluated with respect to their charge mobilities [35,37]. The first report discloses one of the first mobility values measured within the field-effect transistor configuration for P3HT (M_w_ = 39,600 g/mol) single crystals fabricated by controlled solvent vapor annealing and comprising backbones displaced parallel to the substrate while the *π*-*π* stacking direction was orthogonal with respect to the long crystal axis [35]. Here, a modest mobility of 1.57 × 10^−3^ cm^2^V^−1^s^−1^ was determined when the current flowed along the crystal axis. The second report discusses anisotropic transport in large yet highly-ordered oligothiophene (M_n_ = 1332 g/mol) single crystals obtained by self-seeding followed by crystallization at rather elevated temperatures [37]. Using a conductive atomic force microscope (C-AFM), Hourani et al. measured limited mobility in the order of 10^−3^ cm^2^V^−1^s^−1^ in the *π*-*π* stacking direction and along the long axis of a crystal that was driven by a current manifesting space-charge-limited current behavior (Figure 6). Fortunately, a much higher degree of mobility in the order of 0.5 cm^2^V^−1^s^−1^, driven this time by an exponential dependence of the current in the injection mode, was further revealed along the molecular axis and in the direction perpendicular to the crystal’s surface (Figure 6) [37].

Similar charge carrier mobilities were also demonstrated when evaluating single needle-like [36] and rod-like [35] crystals of P3OT systems that were obtained via controlled solvent vapor annealing. While needle-like single crystals comprising longer P3OT molecules packed with their main chains parallel to the long crystal axis and their alkyl side chains oriented orthogonally with respect to the substrate displayed a humble charge carrier mobility of 1.54 × 10^−4^ cm^2^V^−1^s^−1^ in the filed-effect transistor configuration [36], their rod-like single crystal counterparts comprising shorter P3OT chains packed with the π-π stacking direction parallel to the crystal length axis and the main chains parallel to the substrate delivered an important charge mobility of 0.62 cm^2^V^−1^s^−1^ when the current flowed along the long axis of the crystal [35]. Other single crystals made of thiophene-based polymers demonstrated charge carrier mobilities (measured in the filed-effect transistor configuration and along the polymer backbone direction) varying from 0.1 cm^2^V^−1^s^−1^ in the case of TA-PPE (M_w_ = 51,328 g/mol) nanowire crystals [81] to 5.5 cm^2^V^−1^s^−1^ in CDT-BTZ (M_n_ = 50,000 g/mol) fiber-like crystals [82] or to even 7 cm^2^V^−1^s^−1^ in the case of PDTTDPP nanowire-like crystals [79].

**Figure 7:**
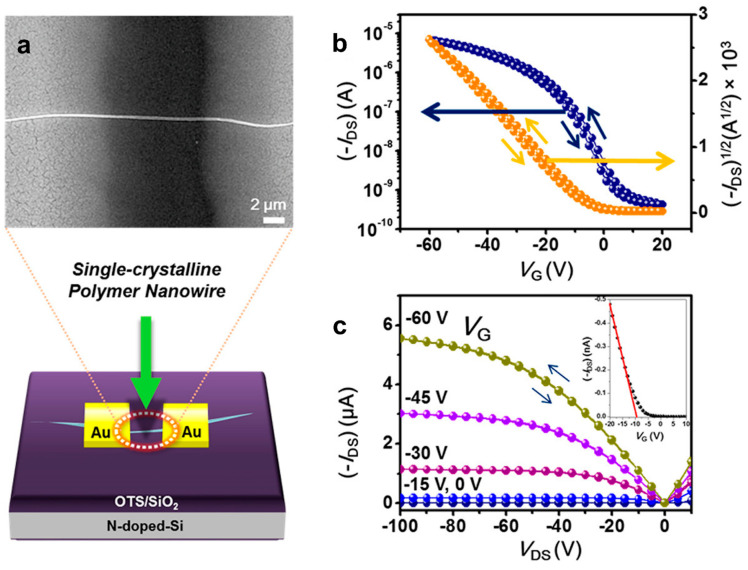
(**a**) SEM micrograph depicting a field-effect transistor device based on a single-crystal DPPBTSPE nanowire (M_n_ = 8000 g/mol), along with schematics illustrating the device configuration. (**b**,**c**) Transfer and output *I–V* curves of the above field-effect transistor device with a drain-to-source voltage of −60 V (with a linear gate voltage range of −5 to −60 V). The inset shows transfer characteristics in the linear regime of the field-effect transistor device based on a single-crystal DPPBTSPE nanowire (with a drain-to-source voltage of −5 V). Adapted from Ref. [80] [copyright (2015), with permission from the American Chemical Society].

It seems that the highest charge carrier mobility measured in a single crystal made of an established semiconducting polymer reached a value of 24 cm^2^V^−1^s^−1^, and it was reported in 2015 by Um et al. for nanowire-like single crystals of rather short DPPBTSPE (M_n_ = 8000 g/mol; Figure 7a) [80]. This astonishing result was achieved by measuring the charge mobility along the direction of the main polymer chains in the filed-effect transistor configuration (Figure 7b,c) and further delivered strong evidence of the importance of intrachain charge transport. Moreover, when longer chains of DPPBTSPE (M_n_ = 68,000 g/mol) were used to fabricate nanowire-like single crystals, charge mobilities only slightly higher than 4 cm^2^V^−1^s^−1^ were obtained, most likely due to the fact that the nanowires were often embedded in an additional network of structures of lower crystallinity [80].

We end this subsection by emphasizing two other high charge-carrier-mobility values (5.58 cm^2^V^−1^s^−1^ and 2.56 cm^2^V^−1^s^−1^) measured very recently in microwire-like crystals made of F4BDOPV-2T (M_n_ = 60,400 g/mol) and P(NDI2OD-T2) (M_n_ = 76,600 g/mol), respectively, by employing a controlled self-seeding technique [87]. In these cases, the reported charge carrier mobilities were measured along the backbones of the polymer chains in field-effect transistor devices. More detailed information on this topic can be found in Table 2.

**Table 2:** Summary of charge carrier mobilities identified in single crystals that were fabricated from established semiconducting polymers.

Polymer	Molecular Weight (g/mol)	Crystal Type	Charge Mobility (cm^2^V^−1^s^−1^)	Current On/Off Ratio	Threshold Voltage (V)	Applications	Ref.
P3HT	M_w_ = 39,600	needle-like	1.57 × 10^−3^	-	-	organic field-effect transistors	[35]
P3HT	M_n_ = 1332	needle-like	0.5	-	8	charge transport studies	[37]
P3OT	M_w_ = 120,000	needle-like	1.54 × 10^−4^	37	7.3	organic field-effect transistors	[36]
P3OT	M_w_ = 51,200	rod-like	0.62	-	-	organic field-effect transistors	[35]
PDTTDPP	-	nanowire	7	-	−15	organic field-effect transistors	[79]
DPPBTSPE	M_n_ = 8000	nanowire	24	10^4^	−4	organic field-effect transistors phototransistors	[80]
DPPBTSPE	M_n_ = 68,000	nanowire	4.15	10^8^	0	organic field-effect transistors phototransistors	[80]
TA-PPE	M_w_ = 51,328	nanowire	0.1	-	−40	organic field-effect transistors	[81]
CDT-BTZ	M_n_ = 50,000	fiber	5.5	10^6^	−60	organic field-effect transistors	[82]
F4BDOPV-2T	M_n_ = 60,400	microwire	5.58	10^3^–10^4^	2	organic field-effect transistors	[87]
P(NDI2OD-T2)	M_n_ = 76,600	microwire	2.56	-	-	organic field-effect transistors	[87]

### 3.2. Conductivity in Single Crystals of Semiconducting Oligomers

**Figure 8:**
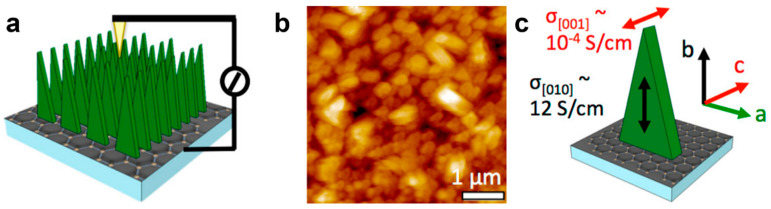
(**a**) Schematics illustrating the experimental C-AFM setup utilized for probing the current of TANI vertical single crystals along the π-stacked *b*-axis. (**b**) AFM image depicting a high density of grown TANI vertical crystals. (**c**) Different conductivities measured along the two crystallographic *b*- and *c*-axes. Adapted from Ref. [86] [copyright (2015), with permission from the American Chemical Society].

A rather unexpected observation can be made right away when discussing the conductivity of single crystals of established semiconducting polymers and oligomers, and that is the high scarcity of research works able to determine the value of this optoelectronic characteristic (note that in general, the bulk conductivity of a semiconducting polymeric material is obtained and dictated by its transport properties along a macromolecular chain as well as between such chains; generally, conductivity depends on the type of charge carriers, their concentrations, and their mobility). In fact, we have identified only one such example in the literature in which a value for conductivity was clearly measured, and it not a polymer but rather the short oligomer TANI [86]. As discussed in Section 2.3, the plate-like TANI single crystals grown were vertically oriented with respect to the graphene guiding substrate. In these crystals, the highest conductivity of 12.3 S/cm was measured with the aid of a C-AFM along the π-stacked *b*-axis (see the experimental configuration utilized as well the evaluated TANI single crystals in Figure 8a,b; similar crystals are also depicted in Figure 5d) [86]. This value was more than ten times higher than the highest conductivity measured previously for tetraaniline in wire, ribbon, or plate-like structures that were previously obtained by Wang et al. using a solvent-exchange process based on a dopant acid [89]. Although it was found that the conductivity along the TANI backbone represented by the *c*-axis was much lower (Figure 8c), these single crystals were nonetheless regarded as great candidates for novel solar cells, sensors, or supercapacitors.

### 3.3. Photovoltaic Properties of Single Crystals of Semiconducting Polymers

**Figure 9:**
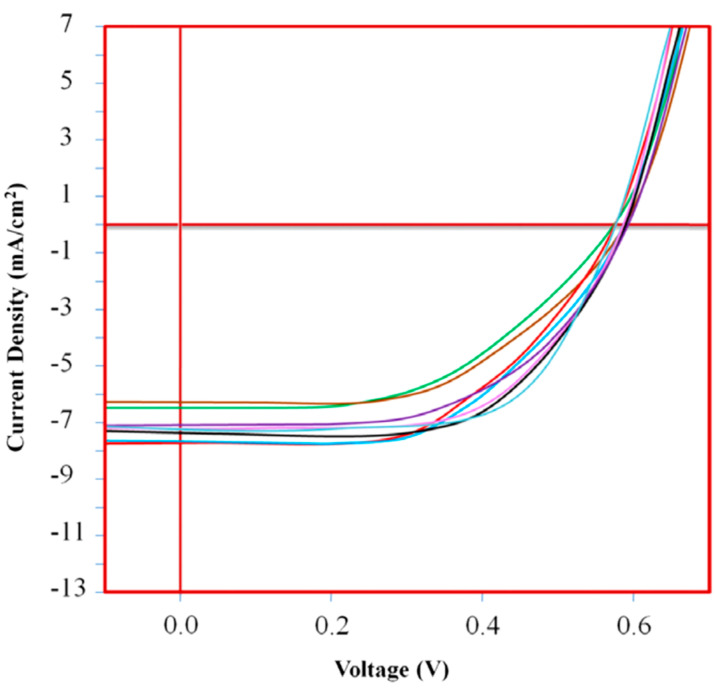
Current–voltage curves of photovoltaic devices based on various active materials comprising the electron acceptor PC71BM and single crystals of P3HT of M_n_ = 7150 g/mol (dark blue; crystals were obtained from toluene), P3HT-*b*-PS of M_n_ = 7669 g/mol (red; crystals were grown from toluene), P3HT of M_n_ = 7150 g/mol (pink; crystals were grown from xylene), P3HT-*b*-PS of M_n_ = 7669 g/mol (black; crystals were obtained from xylene), P3HT of M_n_ = 48,800 g/mol (purple, crystals were grown from toluene), P3HT-*b*-PEG of M_n_ = 49,550 g/mol (light blue; nanofibers were grown from toluene), P3HT of M_n_ = 48,800 g/mol (brown; nanofibers were grown from toluene), and P3HT48800-*b*-PEG of M_n_ = 49,550 g/mol (green; nanofibers were obtained from xylene). Reproduced from Ref. [77] [copyright (2017), with permission from the Polymer Society of Korea and Springer Nature].

There are rather few cases of single crystals based on semiconducting polymers and block copolymers that were studied with respect to their photovoltaic properties [77]. Such crystals were fabricated from various P3HTs, P3HT-*b*-PEG, P3HT-*b*-PS, and P3HT-*b*-PMMA systems and displayed both needle-like and nanofibrillar shapes. When combined with the electron acceptor phenyl-C71-butyric acid methyl ester (PC71BM), these crystals led, without any treatments, to solar cells with a modest power conversion efficiency (PCE) that ranged from 1.49%, corresponding to hairy nanofibers of rather short P3HT-*b*-PMMA chains (M_n_ = 7647 g/mol), to 2.93%, corresponding to needle-like crystals made of longer P3HT chains (M_n_ = 48,800 g/mol; see Table 3). Interestingly, while a maximum short-circuit current density (J_SC_) of 9.26 mA/cm^2^ was obtained for hairy needle-like crystals of P3HT-*b*-PEG (M_n_ = 49,550 g/mol) (Figure 9), the best fill factor (FF) of 55% was measured for needle-like single crystals of P3HT (M_n_ = 48,800 g/mol). Moreover, the highest open-circuit voltage (V_OC_) was determined for both needle-like single crystals of P3HT (M_n_ = 7150 g/mol) and nanofibers of P3HT (M_n_ = 48,800 g/mol) [77].

**Table 3:** Summary of various photovoltaic properties displayed by single crystals prepared from solutions of semiconducting polymers in toluene when mixed with electron-acceptor materials such as PC71BM.

Polymer	Molecular Weight (g/mol)	Crystal Type	J_SC_ (mA/cm^2^)	FF (%)	V_OC_ (V)	PCE (%)	Ref.
P3HT	M_n_ = 7150	needle-like	7.69	54	0.59	2.45	[77]
P3HT	M_n_ = 7150	nanofiber	5.93	45	0.57	1.52	[77]
P3HT	M_n_ = 48,800	needle-like	9.18	55	0.58	2.93	[77]
P3HT	M_n_ = 48,800	nanofiber	7.10	47	0.59	1.97	[77]
P3HT-*b*-PEG	M_n_ = 49,550	hairy needle-like	9.26	54	0.58	2.90	[77]
P3HT-*b*-PEG	M_n_ = 49,550	nanofiber	7.11	53	0.58	2.18	[77]
P3HT-*b*-PS	M_n_ = 7669	hairy needle-like	7.74	51	0.58	2.29	[77]
P3HT-*b*-PMMA	M_n_ = 7647	hairy nanofiber	5.47	47	0.58	1.49	[77]

### 3.4. Absorption Properties of Single Crystals of Semiconducting Polymers

In addition to the charge transport and photovoltaic properties discussed in the above subsections, there are other puzzling optoelectronic properties that can be displayed by single crystals fabricated from established semiconducting polymers. One such property is the ability of single crystals to absorb light, and here we aim to discuss two cases that we identified in the literature which reveal the absorption properties of such crystals [38,75]. In the first case, Agbolaghi et al. studied various P3HT-based scrolled and flat crystalline structures that included fibrillar single crystals prepared via seeded and unseeded protocols involving the isothermal crystallization process [75]. They found that P3HT chains exhibited interesting absorption properties when arranged in highly ordered fibrillar single crystals of P3HT (M_n_ = 7000 g/mol) and P3HT-*b*-PEG (M_n_ = 12,000 g/mol). More precisely, the more ordered P3HT single crystals displayed absorption spectra of slightly higher intensities that were also redshifted with respect to the corresponding spectra of P3HT-*b*-PEG single crystals. Note that both types of crystals were obtained by utilizing seeding protocols. Moreover, when compared to unseeded P3HT and P3HT-*b*-PEG nanofibers prepared via isothermal crystallization, both types of seeded single crystals showed redshifted absorption spectra of significantly higher intensities, most probably due to their higher internal ordering [75]. Furthermore, while similar observations were also made for the obtained half-ring-like P3HT crystalline microstructures, it was concluded that additional arrangements of PEG blocks in such crystalline entities had no impact on their absorption properties. Finally, the least intensified and most blueshifted absorption spectra were recorded for crystalline microstructures comprising P3HT backbones that were simply grafted as conductive brushes on substrates covered with PEG chains [75].

**Figure 10:**
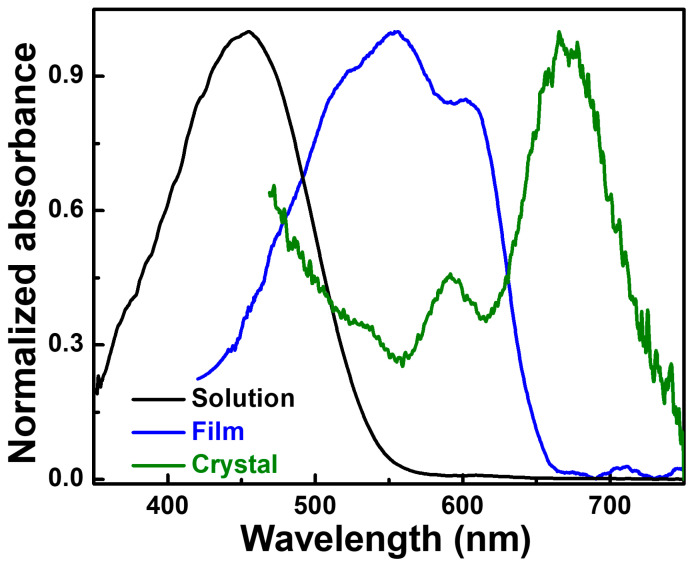
Normalized UV–vis absorption spectra recorded for P3HT chains homogeneously dispersed in a 3-hexylthiophene solution at 50 °C (black), for a P3HT thin film spin-coated from a solution in 3-hexylthiophene (blue) and for a P3HT single crystal spin-coated from a 3-hexylthiophene dispersion previously obtained by self-seeding and subsequent crystallization (olive). Adapted from Ref. [38]—published by The Royal Society of Chemistry.

In the second case, Rahimi et al. discussed the absorption properties of P3HT single crystals obtained by self-seeding combined with crystallization in dilute solutions at rather high temperatures [38]. These single crystals were characterized by high levels of internal order, with P3HT chains (M_n_ = 26,400 g/mol) being fully extended and orthogonally oriented with respect to the substrate (here, all side chains adopted a highly interdigitated conformation) [25,38]. By comparing the absorption spectra of P3HT chains in a solution, thin film, and single crystals, they found that while the spectrum recorded for the solution displayed a single broad peak at the short wavelength of 455 nm corresponding to intrachain interactions between the flexible chains of a random coiled-like conformation, and while the spectrum measured for the thin film exhibited two peaks at wavelengths of 555 nm (A_0-1_) and 603 nm (A_0-0_) characteristic of interchain transitions found in partially ordered H-aggregated chains mixed with non-aggregated chains, the spectrum obtained for the single crystals was significantly redshifted (~70 nm with respect to the film) and displayed an A_0-0_/A_0-1_ intensity ratio much larger than 1 (Figure 10) [38]. This puzzling observation was suggested to be due to long-range intrachain interactions taking place along the fully extended and closely packed P3HT chains experiencing no structural defects or amorphous/coiled-like conformations [38]. While the latter conformations are known to be characterized by low intrachain order and disclose broadened and blueshifted absorption signatures, it was suggested that the closely packed conformations adopted by the P3HT chains in single crystals could experience a double delocalization along and in between the chains [38].

## 4. Conclusions and Perspectives 

One of the great advantages exhibited by single crystals made of semiconducting polymers is the high internal/structural order characterized by a unique molecular conformation adopted by all chains forming such a crystalline entity. Therefore, single crystals are regarded as defect-free with no grain boundaries and are thus ideal structures in which to study, for instance, interchain and intrachain charge transport properties in a perspicuous manner. As a consequence, the scientific community designed and developed a variety of fabrication methods that can deliver large yet highly ordered single crystals of semiconducting polymers and oligomers, including controlled solvent annealing and self-seeding combined with specific crystallization processes. Nonetheless, one has to understand that such fabrication methods imply a rather high level of experimental difficulty mainly generated by complex nucleation and growth kinetics or other limiting processes such as chain entanglements, chain diffusion, and adsorption or desorption. Thus, the number of works reporting single crystals of semiconducting polymers is, at the moment, rather limited.

Nonetheless, based on most of the reports found in the literature, we have concluded that although the internal structures of single crystals are generally well assessed by means of various X-ray and electron diffraction techniques, additional information on the types of crystalline unit cells characterizing the reported crystalline entities is rather scarce (see Table 1). Within this scarcity, most of the work analyzed reveal that semiconducting polymer chains (such as P3HT, PDTTDPP, DPPBTSPE, TA-PPE, F16, F32, and F64) forming single crystals prefer to pack into an orthorhombic unit cell, with the only monoclinic unit cell being reported by Rahimi et al. for self-seeded single crystals of P3HT in Ref. [25]. Moreover, there are only two cases of triclinic unit cells that were preferred by polymer chains such as F4BDOPV-2T and P(NDI2OD-T2) (see Ref. [87]).

Our final remark is on charge transport studies conducted on single crystals of established semiconducting polymers. While there is a rather consistent number of works revealing various values for the charge carrier mobility, measured mainly in the field-effect transistor configuration (with the highest charge mobility of 24 cm^2^V^−1^s^−1^ spotted for single-crystal nanowires of DPPBTSPE, Ref. [80]), there are only very few studies in the literature clearly determining the conductivity (one single conductivity value of 12.3 S/cm was identified for TANI single crystals, Ref. [86]), photovoltaic capability, or light absorption ability of single crystals. Therefore, future studies targeting the above-identified insufficiencies would be of significant importance for the healthy development of single crystals of semiconducting polymers and their desirable applications.
